# Palmitoylation of Prolactin-Releasing Peptide Increased Affinity for and Activation of the GPR10, NPFF-R2 and NPFF-R1 Receptors: In Vitro Study

**DOI:** 10.3390/ijms22168904

**Published:** 2021-08-18

**Authors:** Alena Karnošová, Veronika Strnadová, Lucie Holá, Blanka Železná, Jaroslav Kuneš, Lenka Maletínská

**Affiliations:** 1 Biochemistry and Molecular Biology, Institute of Organic Chemistry and Biochemistry of the Czech Academy of Sciences, 16610 Prague, Czech Republic; alena.karnosova@uochb.cas.cz (A.K.); veronika.strnadova@uochb.cas.cz (V.S.); lucie.cerna@uochb.cas.cz (L.H.); zelezna@uochb.cas.cz (B.Ž.); kunes@biomed.cas.cz (J.K.); 2 First Faculty of Medicine, Charles University, 12108 Prague, Czech Republic; 3 Experimental Hypertension, Institute of Physiology of the Czech Academy of Sciences, 14200 Prague, Czech Republic

**Keywords:** prolactin-releasing peptide, GPR10, neuropeptide FF, NPFF-R2, NPFF-R1, binding properties, signaling pathways

## Abstract

The anorexigenic neuropeptide prolactin-releasing peptide (PrRP) is involved in the regulation of food intake and energy expenditure. Lipidization of PrRP stabilizes the peptide, facilitates central effect after peripheral administration and increases its affinity for its receptor, GPR10, and for the neuropeptide FF (NPFF) receptor NPFF-R2. The two most potent palmitoylated analogs with anorectic effects in mice, palm^11^-PrRP31 and palm-PrRP31, were studied in vitro to determine their agonist/antagonist properties and mechanism of action on GPR10, NPFF-R2 and other potential off-target receptors related to energy homeostasis. Palmitoylation of both PrRP31 analogs increased the binding properties of PrRP31 to anorexigenic receptors GPR10 and NPFF-R2 and resulted in a high affinity for another NPFF receptor, NPFF-R1. Moreover, in CHO-K1 cells expressing GPR10, NPFF-R2 or NPFF-R1, palm^11^-PrRP and palm-PrRP significantly increased the phosphorylation of extracellular signal-regulated kinase (ERK), protein kinase B (Akt) and cAMP-responsive element-binding protein (CREB). Palm^11^-PrRP31, unlike palm-PrRP31, did not activate either c-Jun *N*-terminal kinase (JNK), p38, c-Jun, c-Fos or CREB pathways in cells expressing NPFF-1R. Palm-PrRP31 also has higher binding affinities for off-target receptors, namely, the ghrelin, opioid (KOR, MOR, DOR and OPR-L1) and neuropeptide Y (Y_1_, Y_2_ and Y_5_) receptors. Palm^11^-PrRP31 exhibited fewer off-target activities; therefore, it has a higher potential to be used as an anti-obesity drug with anorectic effects.

## 1. Introduction

Prolactin-releasing peptide (PrRP) was discovered as an endogenous ligand of the orphan G-protein coupled receptor GPR10 (also known as hGR3) in the hypothalamus and has been suggested to stimulate prolactin secretion [1,2]. However, soon after this finding, Lawrence et al. showed a reduction in food intake and body weight and an increase in energy expenditure after intracerebroventricular (ICV) PrRP injection in rats and questioned the role of PrRP in prolactin secretion [3,4]. The effects of PrRP, mostly mediated through the GPR10 receptor, which is widely expressed throughout the brain mainly in areas related to the regulation of food intake and energy homeostasis, confirm GPR10 knockout (KO) mouse studies showing an increase in body weight in KO mice [5,6,7].

PrRP occurs in two biologically active isoforms, PrRP31 and PrRP20. Our previous studies showed the induction of central c-Fos activation of regions related to food intake after peripheral administration of PrRP31 or PrRP20 modified with either myristoyl or palmitoyl, but this central effect was not observed after peripheral administration of natural PrRP31 or PrRP20. Lipidized PrRP31 and PrRP20 analogs decrease food intake and body weight in mice, increase stability and prolong half-life compared to natural peptides [8,9,10,11,12]. PrRP20 and PrRP31 also strongly interact with the receptor of neuropeptide FF (NPFF), NPFF-R2 [13]. Lipidization of PrRP20 and PrRP31 increases in vitro binding affinities not only to GPR10 but also to NPFF-R2 [8,9]. However, lipidized PrRP20 showed lower solubility and bioavailability [8]; therefore, our further studies were focused on lipidized PrRP31 analogs.

PrRP, together with NPFF, belongs to the RF-amide peptide family, which contains a typical *C*-terminal amino acid sequence motif (RF-NH_2_) essential for receptor activation. All RF-amide peptides have a high affinity for and activity on both NPFF receptors NPFF-R2 and NPFF-R1 and may also exert in vivo effects through these receptors [14]. Expression of both NPFF receptors has been found in hypothalamic areas that regulate feeding and energy homeostasis. Moreover, the ability of NPFF to regulate food intake was previously demonstrated, when ICV administration of NPFF was shown to result in decreased food intake in fasted rats [15,16].

Both NPFF receptors show the ability to regulate the cardiovascular system and modulate pain perceptions [17,18,19]. Despite the fact that antagonist of NPFF-R1 and NPFF-R2 RF9 prevents opioid-induced hyperalgesia and that NPFF induces an increase in arterial blood pressure in rats [20], our previous study did not prove the antagonistic activity of RF9 on NPFF-induced anorexigenic effects [21]. Conversely, RF9 exhibits an anorectic effect after ICV or subcutaneous administration in fasted mice [21].

Similar to NPFF, PrRP also appears to have antinociceptive properties [22,23]. Although PrRP has a high affinity for NPFF receptors, its ability to modulate pain perception through NPFF-1R and NPFF-2R has not been proven. Kalliomäki et al. studied the nociceptive properties of 1DMe, a stable NPFF analog, and PrRP in the central nervous system of rats and refuted the ability of PrRPs to regulate pain perception through NPFF receptors [22].

Many G-protein coupled receptors (GPCRs) share similar characteristic features. Receptors GPR10, NPFF-R1 and NPFF-R2 are members of the β-type rhodopsin GPCR family, which has important roles in the regulation of food intake and energy homeostasis [24]. GPR10 has a high percentage of amino sequence identity, especially in the transmembrane regions, with neuropeptide Y receptors, members of the β-type rhodopsin GPCR family, which are involved in food intake regulation [25]. Furthermore, Y receptors share a high percentage of amino sequence homology with NPFF-R1 and NPFF-R2 [26].

The mechanism of action of PrRP is not yet fully understood. PrRP31 and PrRP20 have been shown to mobilize Ca^2+^ from intracellular stores via GPR10 by activating the second messenger IP3 (inositol-1,4,5-trisphosphate), leading to an increase in cytoplasmic Ca^2+^ [27,28], which can subsequently activate the extracellular signal-regulated kinase (ERK) signaling cascade [29]. PrRPs displayed the ability to activate the phosphorylation of ERK, the c-Jun *N*-terminal kinase (JNK) pathway, the cAMP-responsive element binding protein (CREB) pathway and the protein kinase B (Akt/PKB) pathway, which plays a key role in the regulation of protein synthesis and maintenance of glucose homeostasis [30,31,32].

Maixnerová et al. previously showed that the first 20 amino acids of PrRP31 are important for the preservation of full in vivo activity [31]. This study compares the activity of two most potent PrRP31 analogs, palm^11^-PrRP31 and palm-PrRP31, which contain palmitic acid attached to the N-terminus of the amino acid chain (palm-PrRP31) or to the position 11, where original Arg^11^ was replaced with Lys^11^ (palm^11^-PrRP31) (Table 1). These analogs previously showed the ability to significantly decrease food intake and body weight after repeated peripheral administration [8,9], but the mechanism of action is still unclear. We aimed to identify the off-target activity of palm^11^-PrRP31 and palm-PrRP31 to map the mechanism of action and to compare intracellular transduction pathways of anorexigenic receptors GPR10, NPFF-R2, and new strong target of PrRP31 analogs, NPFF-R1. GPR10 is a highly selective receptor for PrRP31 and analogs related to PrRP31. To control the selectivity of PrRP31 for GPR10s, we used NPFF and its stable analog 1DMe in this study. To determine whether the possible analgesic effect of PrRPs is caused by off-target activity, opioid receptors were investigated.

## 2. Results

### 2.1. Binding Affinity for GPR10, NPFF-R2 and Potential Off-Target Receptors

#### 2.1.1. Palmitoylated PrRP31 Analogs Have a High Binding Affinity for GPR10, NPFF-R2 and NPFF-R1

Based on previously published data, affinity for the GPR10 and NPFF-R2 of PrRP31 and its analogs was studied [8,9]. PrRP31 and its two palmitoylated analogs of PrRP31 (see Table 1 for structures) have a high binding affinity for the GPR10 and NPFF-R2 receptors, and their K_i_ values were in the nanomolar range (Table 2). Compared to natural PrRP31, palmitoylated analogs had a higher binding affinity for both of these receptors. Palm^11^-PrRP31 showed a higher affinity for the receptor GPR10 than for the receptor NPFF-R2. NPFF and its stable analog 1DMe displayed negligible affinity for the GPR10 receptor. The affinities of NPFF and 1DMe to NPFF-R2 were detected to be in the nanomolar range (Table 2).

To find another possible target of the two most potent palmitoylated analogs of PrRP31, binding to NPFF-R1 was tested. Membranes from CHO-K1 cells expressing the NPFF-R1 were isolated, and the K_d_ was determined to be 0.94 ± 0.06 nM by saturation experiments using the radioligand [^125^I]-1DMe. Although natural PrRP31 bound to NPFF-R1 with a lower affinity than to NPFF-R2, the binding affinity was still in the 10^−8^ M range (Table 2). Palmitoylation increased the binding affinities of both analogs to NPFF-R1. Palm-PrRP31 showed binding affinities in the nanomolar range to both NPFF receptors compared to palm^11^-PrRP31 (Table 2).

#### 2.1.2. Palm-PrRP31 Shows a Higher Affinity for Other Potential Off-Target Receptors than Palm^11^-PrRP31

Several other potential off-target receptors of PrRP31 and its palmitoylated analogs were tested. The binding properties of PrRP31, palm^11^-PrRP and palm-PrRP31 to receptors Y_1_, Y_2_, and Y_5_, ghrelin receptor (also growth hormone secretagogue receptor—GHSR) and kappa-opioid receptor (KOR) were determined. The natural ligand PYY of Y receptors bound in the nanomolar range to the Y_1_, Y_2_, and Y_5_ receptors (Table 2). From saturation binding experiments with [^125^I]-PYY as a radioligand, the K_d_ for each receptor was determined. The K_d_ for Y_1_ was 1.53 ± 0.08 nM, for Y_2_ was 2.18 ± 0.85 nM and for Y_5_ was 1.01 ± 0.27 nM. Natural PrRP31 had no affinity to the Y_1_ and Y_2_ receptors in the range of measured concentrations, but it showed a very low affinity to the Y_5_ receptor. Compared to palm^11^-PrRP31, palm-PrRP31 exhibited a relatively high affinity for the Y_5_ receptor. Both palmitoylated analogs bound to Y_1_ and Y_2_ with a negligible low affinity (Table 2).

The K_d_ determined by a saturation binding experiment with [^125^I]-dynorphin as a radioligand was 2.38 nM. The agonist dynorphin showed a very high affinity for the KOR receptor, but no binding was observed with natural PrRP31 (Table 2). Palmitoylation enhanced binding to the KOR receptor. Palm-PrRP31 bound to KOR with a higher affinity than palm^11^-PrRP31, but both were in the 10^−7^–10^−6^ M range.

Another tested potential off-target receptor was the ghrelin receptor GHSR. From saturation experiments using [^125^I]-ghrelin as a radioligand, a K_d_ of 0.44 ± 0.12 nM was determined. Natural PrRP31 showed no binding to GHSR in competitive binding experiments in the measured range, but palmitoylated analogs showed a low binding affinity for this receptor (Table 2). Palm-PrRP31 had a higher affinity for GHSR than palm^11^-PrRP31.

### 2.2. PrRP31 and Its Palmitoylated Analogs Stimulate Ca^2+^ Mobilization in CHO-K1 Cells Expressing GPR10 or NPFF-R2

**Figure 1:**
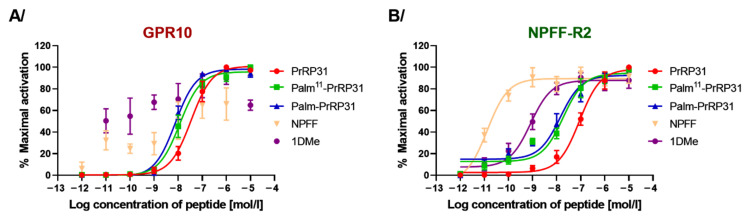
Intracellular Ca^2+^ mobilization in CHO-K1 cells expressing (**A**) GPR10 or (**B**) NPFF-R2. Data are presented as mean ± SEM, and the experiments were performed in duplicates and repeated two (GPR10) or three (NPFF-R2) times in duplicates.

Stimulation of Ca^2+^ in CHO-K1 cells expressing the GPR10 receptor was monitored using the calcium-sensitive dye Fura 2. No calcium mobilization was observed after stimulation with the NPFF-R2 agonists NPFF and 1DMe (Figure 1). On the other hand, natural PrRP31, palm^11^-PrRP31 and palm-PrRP31 stimulated Ca^2+^ mobilization. Both of the lipidized analogs showed a similar Ca^2+^ release response, which was observed at lower concentrations compared to PrRP31.

The CHO-K1 cell line expressing NPFF-R2 with aequorin protein, which detects intracellular Ca^2+^ release, was used to study the agonist properties of PrRP31, its palmitoylated analogs, NPFF and 1DMe. NPFF (EC_50_, 0.24 ± 0.02 pM) and 1DMe (EC_50_, 0.82 ± 0.15 nM) stimulated intracellular Ca^2+^ release at much lower concentrations than the GPR10 agonist PrRP31 (EC_50_, 89.33 ± 0.84 nM) and its lipidized analogs. Palmitoylation of PrRP31 increased agonist activity at NPFF-R2, where the EC_50_ of palm^11^-PrRP31 was 18.71 ± 1.31 nM and that of palm-PrRP31 was 14.16 ± 1.52 nM (Figure 1).

### 2.3. Palmitoylated PrRP31 Analogs Activate Different Intracellular Signaling Pathways in GPR10-, NPFF-R2- or NPFF-R1-Expressing Cells

**Figure 2:**
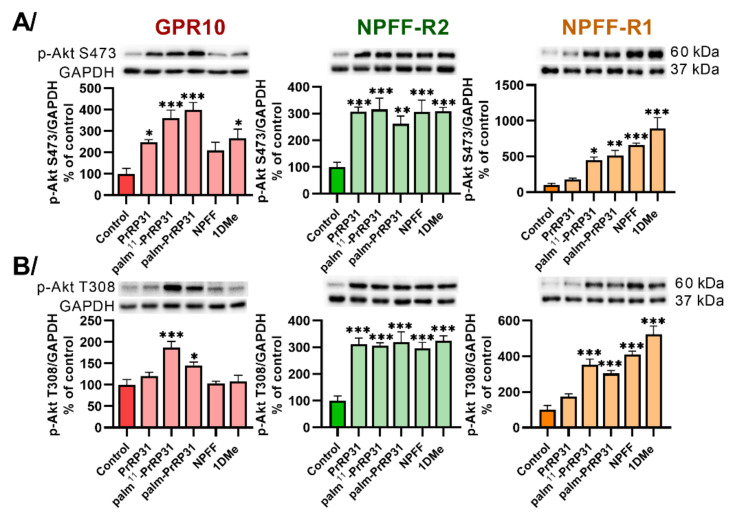
Induction of (**A**) Akt (S473) and (**B**) Akt (T308) phosphorylation after 5 min of incubation at 37 °C with peptides at final concentrations of 10^−6^ M in CHO-K1 cells expressing receptors GPR10, NPFF-R2 and NPFF-R1. Densitometric quantification was normalized to GAPDH, and the phosphorylation level in the untreated control was standardized as 100%. Data are presented as the mean ± SEM and analyzed by two-way ANOVA followed by Dunnett’s post hoc test. Experiments were performed independently at least three times. Statistically significant differences from the control are indicated (* *p* < 0.05, ** *p* < 0.01, *** *p* < 0.001).

**Figure 3:**
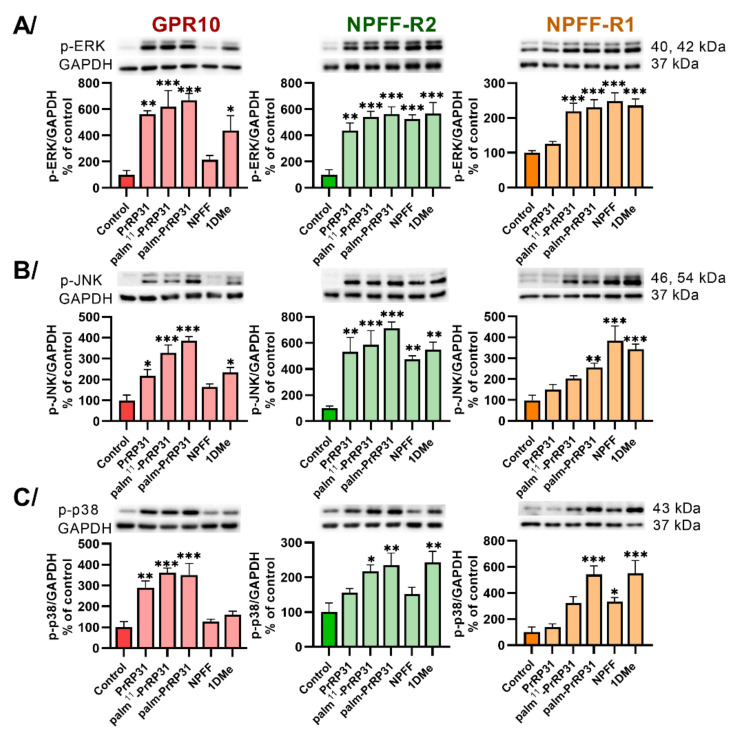
Induction of MAPK pathways: phosphorylation of (**A**) ERK and (**B**) JNK after 5 min and (**C**) p38 after 60 min of incubation at 37 °C with peptides at final concentrations of 10^−6^ M in CHO-K1 cells expressing receptors GPR10, NPFF-R2 and NPFF-R1. Densitometric quantification was normalized to GAPDH, and the phosphorylation level in the untreated control was standardized as 100%. Data are presented as the mean ± SEM and analyzed by two-way ANOVA followed by Dunnett’s post hoc test. Experiments were performed independently at least three times. Statistically significant differences from the control are indicated (* *p* < 0.05, ** *p* < 0.01, *** *p* < 0.001).

**Figure 4:**
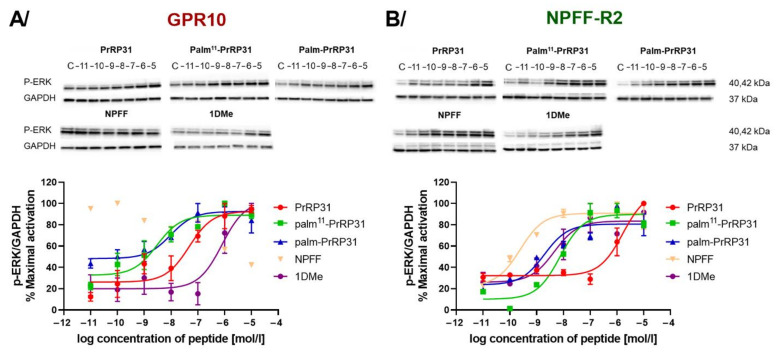
Dose-response phosphorylation of ERK in CHO-K1 cells expressing (**A**) GPR10 and (**B**) NPFF-R2 after 5 min of incubation at 37 °C with peptides at final concentrations from 10^−11^ to 10^−5^ M. Densitometric quantification was normalized to GAPDH. Data are presented as the mean ± SEM, and the experiments were performed independently at least two times and were analyzed using nonlinear regression.

**Figure 5:**
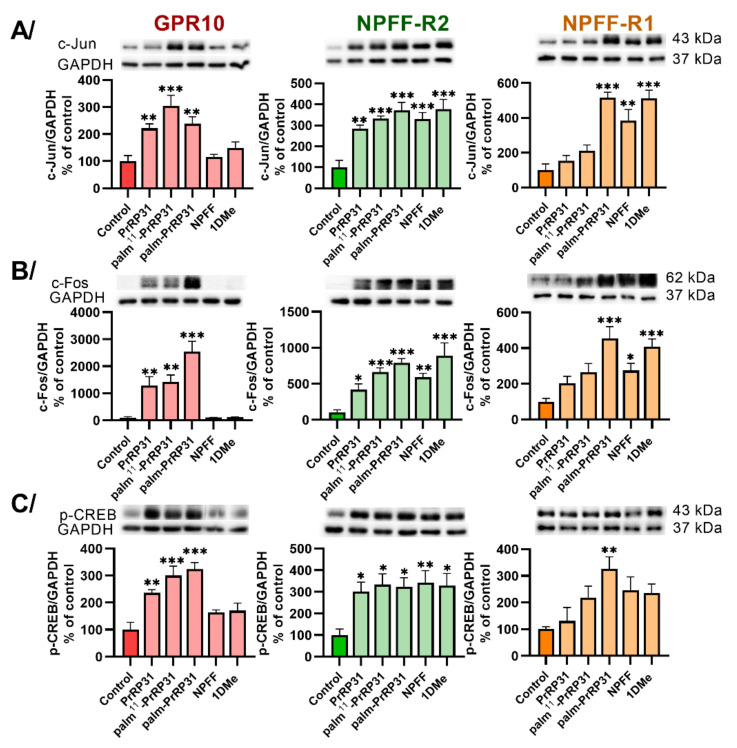
Activation of (**A**) c-Jun and (**B**) c-Fos after 60 min incubation and induction of (**C**) CREB phosphorylation after 5 min incubation at 37 °C with peptides in final concentrations 10^−6^ M in CHO-K1 cells expressing receptors GPR10, NPFF-R2 and NPFF-R1. Densitometric quantification was normalized to GAPDH and the phosphorylation level in the untreated control was standardized as 100%. Data are presented as mean ± SEM and analyzed by two-way ANOVA followed by Dunnett’s post hoc test. Experiments were performed independently at least three times. Statistically significant differences from the control are indicated (* *p* < 0.05, ** *p* < 0.01, *** *p* < 0.001).

To determine the intracellular mechanism of action of PrRP31 and its palmitoylated analogs, several signaling pathways were tested in cells expressing GPR10, NPFF-R2 or NPFF-R1 receptors using immunoblotting (Figure 2, Figure 3, Figure 4 and Figure 5; Supplementary Figure S1). No changes in total protein levels were observed (Supplementary Figure S1B); therefore, only activated/phosphorylated proteins were quantified and compared. NPFF and 1DMe were used as negative controls to validate GPR10 selective properties.

To study PKB/Akt pathway activation, phosphorylation of Akt at Ser473 (Figure 2A) and Thr308 (Figure 2B) was tested. Both PrRP31 analogs, palm^11^-PrRP31 and palm-PrRP31, showed significantly increased phosphorylation of Akt at Ser473 (Figure 2A) and Thr308 (Figure 2B) in cells with GPR10 and NPFF-R2 but also in cells expressing NPFF-R1. Natural PrRP31 did not significantly activate Akt (Figure 2) in cells with NPFF-R1. NPFF and 1DMe increased the phosphorylation of Akt at either Ser473 (Figure 2A) or Thr308 (Figure 2B) in cells containing NPFF-R2 and NPFF-R1, but they were less effective at GPR10.

The activation of the cAMP-dependent protein kinase (PKA) was also studied (Supplementary Figure S1A). No significant changes were observed after treatment with PrRP31, palmitoylated PrRP31 analogs, NPFF or 1DMe in cells expressing GPR10, NPFF-R2 and NPFF-R1.

One of the key signaling pathways of GPCR signaling, the mitogen-activated protein kinase (MAPK) pathway, was also studied. The phosphorylation of MAPKs, ERK, JNK and p38 was significantly increased in CHO-K1 cells expressing receptors GPR10, NPFF-R2 and NPFF-R1 after stimulation with palm-PrRP31 (Figure 3A–C). Palm^11^-PrRP31 significantly increased the phosphorylation of ERK, JNK and p38 in CHO-K1 cells expressing GPR10 and NPFF-R2 (Figure 3A–C), but no significant increase in JNK and p38 was observed in cells with NPFF-R1 (Figure 3B,C). Natural PrRP31 was effective in cells expressing GPR10 and NPFF-R2 but did not activate ERK (Figure 3A), JNK (Figure 3B) or p38 (Figure 3C) in cells transfected with NPFF-R1.

To further characterize the signaling of receptors GPR10 and NPFF-R2, dose-response experiments were performed. The EC_50_ of ERK activation in cells expressing GPR10 was in the nanomolar range after stimulation with PrRP31, palm^11^-PrRP and palm-PrRP31 (Figure 4A). Cells expressing NPFF-R2 showed a strong response with EC_50_ in nanomolar concentrations after stimulation with natural PrRP31, palmitoylated PrRP31 analogs, NPFF or 1DMe (Figure 4B).

Finally, three DNA-binding proteins, cyclic AMP-responsive element-binding (CREB), c-Jun and c-Fos protein, which activate transcription factors, were tested (Figure 5). Palm-PrRP31 significantly increased the activation of c-Jun (Figure 5A) and c-Fos (Figure 5B) and the phosphorylation of CREB (Figure 5C) compared to the nontreated control in the CHO-K1 cells expressing GPR10, NPFF-R2 or NPFF-R1. Stimulation with palm^11^-PrRP31 significantly increased the activation of all three DNA-binding proteins (Figure 5) in cells with GPR10 and NPFF-R2, but was ineffective in cells expressing the NPFF-R1 receptor. No activation in GPR10 after stimulation with NPFF and its stable analog 1DMe was observed, unlike in NPFF-R2 or NPFF-R1, where significantly increased activation was monitored.

The results showing signaling pathway activation determined using immunoblotting in CHO-K1 cells expressing GPR10, NPFF-R2 and NPFF-R1 incubated with peptides at final concentrations of 10^−6^ M are summarized in Table 3.

### 2.4. Agonist and Antagonist Properties of PrRP31 and Its Palmitoylated Analogs at Other Potential Off-Target Receptors

Using the beta-lactamase reporter gene assay with a FRET substrate, receptor activation was studied to establish agonist and antagonist properties of natural PrRP31 and palmitoylated PrRP31 analogs.

Both tested palmitoylated PrRP analogs were strong agonists of the GPR10 receptor, and their EC_50_ values were in the picomolar range (Table 4). Palm^11^-PrRP31 had stronger agonist activity on GPR10 than the analog palm-PrRP31.

Natural PrRP31 was not effective at any tested possible off-target receptor. Both palm^11^-PrRP31 and palm-PrRP31 did not show any agonist activity on the DOR, MOR and ORL-1 opioid receptors, but they did have very weak agonist activity on the KOR (Table 4). In addition, lipidized analogs exerted weak agonist effects on GHSR.

**Figure 6:**
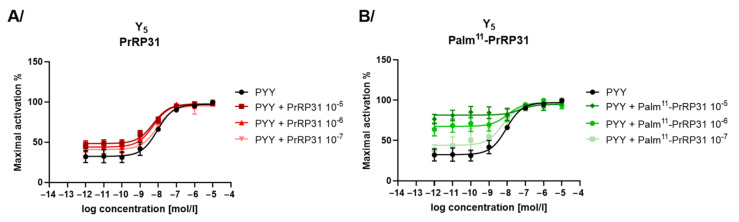
Antagonist mode assay showing effect of (**A**) PrRP31 and (**B**) palm^11^-PrRP31 at Y_5_ receptor together with PYY agonist. Data are presented as mean ± SEM, and the experiments were performed in duplicates and repeated at least two times and analyzed using nonlinear regression.

Compared to palm^11^-PrRP31, palm-PrRP31 showed much stronger agonist activity on GHSR and the Y_5_ receptor (Table 4); therefore, antagonist activity on receptors Y_5_, GHSR and opioid receptors was tested only with PrRP31 and palm^11^-PrRP31. No antagonist properties of PrRP31 and palm^11^-PrRP31 were observed with receptors Y_5_ (Figure 6), GHSR or opioid receptors (KOR, DOR, MOR, ORL-1) (Supplementary Figure S2A,B). Palm^11^-PrRP31 was shown to be a positive allosteric modulator for the Y_5_ receptor, enhancing PYY activity (Figure 6B).

## 3. Discussion

Palmitoylated analogs of neuropeptide PrRP31 previously showed anorexigenic effects and central c-Fos activation after peripheral administration, as well as increased central insulin and leptin signaling, suggesting great potential for the treatment of not only obesity but also neurodegenerative disorders [9,34,35]. PrRP31 has a high affinity for its receptor GPR10, but it also binds to NPFF-R2 [13]. Based on the results of our previous studies the mechanism of action of the two most potent palmitoylated PrRP31 analogs, palm^11^-PrRP31 and palm-PrRP31 on the anorexigenic receptors GPR10 and NPFF-R2 was mapped.

Palmitoylation increased the binding properties of PrRP31 to both of these receptors. Palm^11^-PrRP31 had a higher affinity for the GPR10 receptor than palm-PrRP31, and both analogs displayed an affinity for the NPFF-R2 in the nanomolar range. In this study, several possible off-target receptors of PrR31 were tested. Both of the PrRP31 analogs showed a stronger affinity for the NPFF-R1 than natural PrRP31. Therefore, NPFF-R1 is now considered another relevant target of lipidized PrRP31 analogs.

The activation of intracellular Ca^2+^ mobilization in the CHO-K1 AequoScreen cell line expressing NPFF-R2 showed the agonist properties of PrRP31 and its palmitoylated analogs. Palmitoylation increased the agonist properties of PrRP31 on the receptor NPFF-R2. However, NPFF and its stable analog 1DMe have much stronger agonist activity on its NPFF-R2 receptor than palm^11^-PrRP31 and palm-PrRP31. The activation of GPR10 was studied using the β-lactamase assay with a FRET substrate and a FLIPR calcium assay measuring intracellular Ca^2+^ mobilization. The EC_50_ values of palm^11^-PrRP31 and palm-PrRP31 were in the picomolar range, and the activation was increased three times after palmitoylation. Previous studies suggested that GPR10 is coupled with Gi/o proteins [30,32]. Other studies have shown the ability of PrRP to stimulate cAMP in rat PC12 cells [36] and CHO-K1 cells expressing GPR10 [37], which pointed to Gs protein coupling. However, Langmead et al. revealed the PrRP-induced mobilization of intracellular Ca^2+^ after GPR10 activation, and the PrRP’s inability to suppress cAMP levels after forskolin stimulation in HEK293 cells transfected with GPR10. These results suggested that GPR10 is coupled with the Gq protein [27]. We observed intracellular Ca^2+^ mobilization after stimulation with PrRP31 and its palmitoylated analogs, which may suggest that GPR10 is coupled with either Gi or Gq. This also supported our finding that PKA was not activated after stimulation with PrRP31 and its palmitoylated analogs; thus, GPR10 was not coupled with Gs proteins.

**Figure 7:**
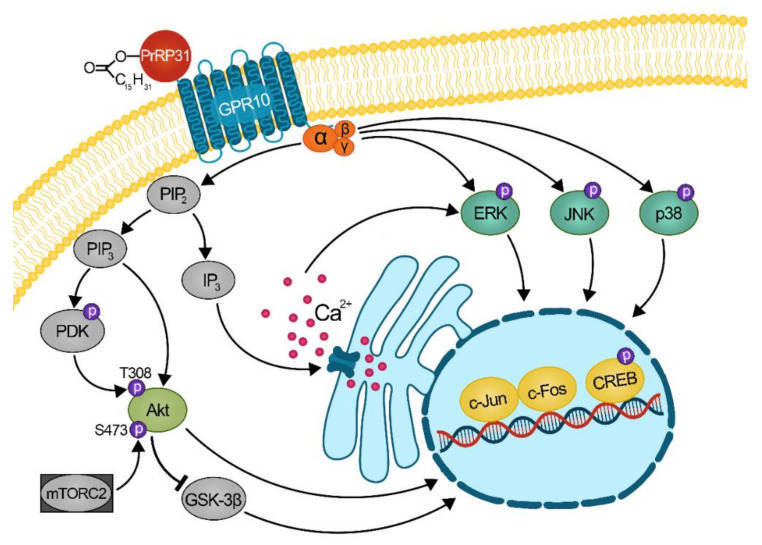
Scheme of mechanism of action of palmitoylated PrRP31 analogs at GPR10: ERK, extracellular signal-regulated kinase; JNK, c-Jun *N*-terminal kinase; CREB, cAMP-responsive element binding protein; PIP_2_, phosphatidylinositol 4,5-bisphosphate; IP_3_, inositol 1,4,5-triphosphate; PIP_3_, phosphatidylinositol (3,4,5)-trisphosphate; PDK, phosphoinositide-dependent kinase 1; Akt, protein kinase B; mTORC2, mammalian target of rapamycin complex 2; GSK-3β, glycogen synthase kinase-3β.

In this study, the intracellular signaling pathways of PrRP31 and its palmitoylated analogs in CHO-K1 cells transfected with GPR10, NPFF-R2, or NPFF-R1 were explored using immunoblotting, and the possible signal transduction of GPR10 was suggested (Figure 7). Haykawa et al. previously showed the activation of Akt in rat pituitary GH3 cells after 5 min of stimulation with PrRP [32]. Our study found significant induction of Akt phosphorylation at T308 and S473 in CHO-K1 cells expressing GPR10, NPFF-R2 and NPFF-R1 after 5 min of stimulation with palm^11^-PrRP31 and palm-PrRP31, but no significantly increased phosphorylation was observed after stimulation with natural PrPR31 in cells with NPFF-R1. Palmitoylation helped stabilize PrRP31 and increased the induction activity of Akt through the receptors GPR10, NPFF-R2 and NPFF-R1. Previous studies demonstrated that PrRP activated the MAP kinases ERK and JNK in rat GH3 cells [30] and PC12 cells [38]. Our results showed significant activation of JNK, ERK and p38 MAPKs after PrRP31 incubation in CHO-K1 cells expressing GPR10, and of JNK and ERK in cells with NPFF-R2. Both palmitoylated PrRP31 analogs also significantly increased the phosphorylation of all three tested MAPKs in GPR10 and NPFF-R2-expressing cells. Dose-response experiments showed the ability of PrRP31, palm^11^-PrRP31 and palm-PrRP31 to activate ERK phosphorylation in cells with GPR10 and NPFF-R2 in the nanomolar range. JNK and ERK activation play important roles in cell proliferation, differentiation and apoptosis by promoting the formation of AP1 complexes, important transcription factors controlling the cell cycle, through the activation of c-Fos and c-Jun [39]. Similar to ERK and JNK, p38 is also connected with cell cycle regulation, regulation of stress responses, immune responses and cell differentiation [40]. Palm^11^-PrRP31 and palm-PrRP31 were found to significantly increase p38 phosphorylation in cells expressing GPR10 and NPFF-R2, and stimulation with palm-PrRP31 induced p38 phosphorylation in cells expressing NPFF-R1. Both inducible transcription factors, c-Fos and c-Jun, were significantly activated after stimulation with PrRP31 and its palmitoylated analogs in cells with GPR10 and NPFF-R2. Conversely, NPFF-R1 significantly activated c-Fos and c-Jun only after stimulation with palm-PrRP31. The transcription factor CREB is also important for the regulation of cell proliferation, cell survival and differentiation, for maintaining glucose homeostasis, and has an important role in activating immune responses [40,41]. Likewise, c-Fos and c-Jun activation and phosphorylation of the transcription factor CREB were significantly increased in cells expressing GPR10 and NPFF-R2 after stimulation with PrRP31 and its analogs. Compared to palm^11^-PrRP31, palm-PrRP31 showed a higher activity in cells transfected with NPFF-R1 in all tested signaling pathways. The results show that PrRP31, palm^11^-PrRP31 and palm-PrRP31 may play important roles in the regulation of cell proliferation and affect immune responses. These findings suggest that dysregulation of glucose homeostasis and inflammatory responses linked with obesity could be treated with PrRP31 analogs.

In this study, we tested potential off-target receptors of PrRP31, which are related to food intake and energy metabolism. Because PrRP and NPFF were found to have antinociceptive properties [16,17,22,23], their agonist and antagonist activities on opioid receptors were studied using a β-lactamase assay. In our study, palmitoylation increased the binding properties of natural PrRP31. Palm^11^-PrRP31 was found to have a lower affinity for KOR than palm-PrRP31, but they both had negligible ability to activate the KOR receptor in either agonist mode or antagonist mode. We did not observe any agonist or antagonist activity of either PrRP31 palmitoylated analog on the other opioid receptors MOR, DOR and ORL-1. The possible pain modulation properties of PrRP do not seem to be linked to opioid receptors, which supports the idea that GPR10 is involved in pain processing regulation. A study by Laurent et al. using GPR10 KO suggested that the central anti-opioid activity of NPFF in mice is regulated by GPR10. Moreover, they suggested that the dual coupling of GPR10 with Gq and Gi may be the reason for PrRP’s involvement in different neuronal networks [23]. GPR10 could be involved either in pain modulation or food intake regulation, depending on the type of G protein coupled with GPR10.

NPY, together with PYY and pancreatic polypeptide (PP), controls energy homeostasis though NPY receptors. NPY receptors are expressed throughout the central nervous system but can also be found in the peripheral nervous system [42]. The affinity of PrRP31 and its palmitoylated analogs for the receptors Y_1_, Y_2_ and Y_5_ was tested. No binding affinity of PrRP31 and palmitoylated analogs for the Y_2_ receptor was observed and a negligible affinity of palm-PrRP31 for Y_1_ was detected. However, natural PrRP31, palm^11^-PrRP31 and palm-PrRP31 bound and activated the Y_5_ receptor with a K_i_ and an EC_50_ in the micromolar range. PrRP31 affinity and agonist activity were increased with the attached palmitoyl group. Y_1_ and Y_5_ receptors are expressed in the same neurons, and they both have important regulatory functions in food intake and energy balance [42]. Although NPY is an orexigenic peptide, Y_1_ and Y_5_ receptor deletion leads to obesity and decreases food intake [43]. This study showed that palm^11^-PrRP31 and palm-PrRP31 had agonist activity ranging from 10^−7^ to 10^−8^ M on the Y_5_ receptor, and palm^11^-PrRP31 was also shown to be a positive allosteric modulator, which suggests that PrRP31 analogs could mediate the in vivo ability to reduce food intake through Y_5_ receptors.

Finally, the off-target properties of the palmitoylated PrRP31 analogs on the receptor of the orexigenic peptide ghrelin were studied. Palm-PrRP31 had a higher affinity for the GHSR receptor than palm^11-^PrRP31, but both analogs had negligible activity on GHSR.

Palm^11^-PrRP31 and palm-PrRP31 displayed higher affinity for GPR10 and NPFF-R2 receptors than natural PrRP31, and stimulation with PrRP31 analogs activated transcription factors c-Fos, c-Jun and CREB and also activated PKB/Akt, MAPK pathways in cells expressing these receptors. A new strong target of palmitoylated analogs was found to be NPFF-R1. Palm-PrRP31 induced activation of tested signaling pathways in cells expressing NPFF-R1. Both analogs revealed negligible affinity and ability to activate receptors Y, opioid receptors and GHSR, but palm-PrRP31 showed higher off-target binding affinity for these possible off-target receptors. Palm^11^-PrRP31 was a more selective agonist of anorexigenic receptors GPR10 and NPFF-R2, with less off-target activity; therefore, it has higher potential for the treatment of obesity and neurodegenerative diseases.

## 4. Materials and Methods

### 4.1. Material

Human PrRP31, palm^11^-PrRP31, palm-PrRP31, neuropeptide FF (NPFF), its stable analog 1DMe (see Table 1 for structures), and ghrelin (ghr) were synthetized and purified as described previously [9,31]. PrRP31 palmitoylation was performed on fully protected peptide on resin as a last step [44]. Peptide purification and identification were determined by analytical high-performance liquid chromatography and by using a Q-TOF micro MS technique (Waters, Milford, MA, USA). Purity of the synthesized peptides was greater than 95%.

Human peptide YY (PYY) (#SC319) was obtained from the PolyPeptide Group (Strasbourg, France). The selective KOR agonists (±)-trans-U-50488 methanesulfonate salt (#D8040) were purchased from Sigma-Aldrich (St. Louis, MS, USA). [D-Pro10]-dynorphin A (#021-17), used as an agonist of KOR in binding experiments, was purchased from Phoenix Pharmaceuticals (Burlingame, CA, USA). Selective agonists of mu-opioid receptor (MOR) DAMGO (#1171), opioid receptor-like 1 (ORL-1) agonist nociceptin (#0910) and agonist of delta-opioid receptor (DOR) [D-Ala2]-deltorphin II (#1180) were obtained from Tocris (Bristol, UK).

### 4.2. Peptide Iodination

Human PrRP31 and 1DMe were iodinated at Tyr20 and D-Tyr1, respectively, with Na[^125^I] purchased from Izotop (Budapest, Hungary) using IODO-GEN (Pierce, Rockford, IL, USA), as described previously [44]. Radioligands [^125^I]-PYY, [^125^I]-dynorphin A and [^125^I]-ghrelin were iodinated at Tyr20 (Tyr27), Tyr1 and His9, respectively. The identity of peptides was determined by a MALDI-TOF Reflex IV mass spectrometer (Bruker Daltonics, Billerica, MA, USA). The specific activity of all ^125^I-labeled peptides was approximately 2100 Ci/mmol. The radiolabeled peptides were kept in aliquots at −20 °C and used in experiments within 1 month.

### 4.3. Cell Cultures

All used cells were maintained at 37 °C in a humified incubator with 5% CO_2_. Growth and assay media were prepared according to manufacturer protocols, and cells were cultured as required. Chinese hamster ovary cells (CHO-K1) stably expressing receptors GPR10 (#K1732) or kappa-opioid receptor (KOR) (#K1533) and human bone osteosarcoma epithelial cells (U2OS) stably expressing receptors of NPY (Y_1_ (#K1803), Y_2_ (#K149), Y_5_ (#K1782)), mu-opioid receptor (MOR) (#K1523), delta-opioid receptor (DOR) (#K1778), opioid receptor-like 1 (ORL-1) (#K1786) and ghrelin receptor (GHSR) (#K1819) were all obtained from Thermo Fisher Scientific Inc. Brand (Waltham, MA, USA). CHO-K1 cell lines containing NPFF-R2 (#ES-490-A) and NPFF-R1 (#ES-491-C) were obtained from Perkin Elmer (Waltham, MA, USA).

### 4.4. Cell Membrane Isolation

Pellets of CHO-K1 cells containing NPFF-R2, NPFF-R1 and KOR receptors were homogenized in ice-cold homogenizing buffer (20 mM HEPES pH 7.1, 5 mM MgCl_2_, 0.7 mM bacitracin) with a DIAX 100 Homogenizer (Heidolph Instruments, Schwabach, Germany) and centrifuged in an ultracentrifuge (Beckman Coulter, Fullerton, CA, USA) at 26,000× *g* for 15 min at 4 °C. The pellets were homogenized in ice-cold homogenization buffer, and the previous steps were repeated 2 more times. After the third centrifugation, pellets were resuspended in ice-cold storage buffer (50 mM Tris-Cl pH 7.4, 0.5 mM EDTA, 10 mM MgCl_2_, 10% sucrose), and aliquots were stored at −80 °C. The concentration of isolated membrane proteins was determined by a PierceTM BCA Protein Assay Kit (Pierce, Rockford, IL, USA).

### 4.5. Competitive Binding Experiments

Competition binding experiments were performed according to [45]. [^125^I]-PrRP31 was used to compete with human PrRP31, palmitoylated PrRP31 analogs, NPFF, and 1DMe in CHO-K1 cells expressing human GPR10 as described previously [31]. Binding experiments using U2OS cells were optimized and performed in assay buffer (50 mM Tris-Cl pH 7.4, 118 mM NaCl, 5 mM MgCl_2_, 4.7 mM KCl, 0.1% BSA) for cells stably expressing GHSR and (25 mM HEPES, 120 mM NaCl, 5 mM MgCl_2_, 4.7 mM KCl, 1 mM CaCl_2_, 0.5% BSA, 2 g/L glucose) for cells containing receptors Y_1_, Y_2_ and Y_5_. PrRP31 or lipidized analogs of PrRP31,ghr, or PYY were used at final concentrations from 10^−12^ to 10^−5^ M to compete with 0.1 nM [^125^I]-ghr, or [^125^I]-PYY radioligands. Cells were incubated for 60 min at room temperature (RT).

Plasma membranes isolated from CHO-K1 cells containing receptors NPFF-R2, NPFF-R1 and KOR were used at a concentration of 5 µg of protein/tube, and binding experiments were performed in assay buffer (50 mM Tris-HCl pH 7.4 + 60 mM NaCl + 1 mM MgCl2 + 0.5% BSA). [^125^I]-1DMe was used to compete with human PrRP31, palmitoylated PrRP31 analogs, NPFF or 1DMe in isolated membranes with NPFF-R2 or NPFF-R1, and [^125^I]-dynorphin A was used to compete with human PrRP31 and palmitoylated PrRP31 analogs in isolated membranes with KOR. The studied peptides and radioligands were incubated with plasma membranes for 60 min at RT and subsequently filtered in a Brandel cell harvester (Biochemical and Development Laboratories, Gaithersburg, MD, USA) using Whatman GF/B filters preincubated in 0.3% polyethylenimine. Filters were rinsed three times with 2 mL of wash buffer (50 mM Tris pH 7.4 + 60 mM NaCl).

Radioactivity was determined by a γ-counter Wizard 1470 Automatic Gamma Counter (Perkin Elmer). Experiments were carried out in duplicate at least three times, and K_i_ was calculated using the Cheng-Prusoff equation.

### 4.6. Cell Signaling Detection by Immunoblotting

Activation of signaling pathways was studied in the CHO-K1 cell lines containing GPR10, NPFF-R2 and NPFF-R1. Cells were seeded in 24-well plates at 30,000 cells/well in assay medium (growth medium without selective antibiotics) and were grown for 2 days. The day before the experiment, the medium was changed to serum-free medium. On the day of the experiment, cells were incubated with PrRP31, lipidized PrRP31 analogs, NPFF or 1DMe at final concentrations from 10^−11^ to 10^−5^ M for 5 min or 60 min at 37 °C and then washed three times with ice-cold phosphate-buffered saline (PBS) pH 7.4. Cells were lysed with Laemmli sample buffer (62.5 mM Tris-HCl at pH 6.8, 2% SDS, 10% glycerol, 0.01% bromophenol blue, 5% β-mercaptoethanol, 50 mM NaF, and 1 mM Na_3_VO_4_). Samples were stored at −20 °C. Electrophoresis and immunoblotting were performed as described previously [45]. For detection of signaling pathways, primary monoclonal antibodies (see Table 5 for the antibodies used) purchased from Cell Signaling Technology (Danvers, MA, USA) were used.

### 4.7. Calcium Mobilization Assays

Measuring the intracellular Ca^2+^ level in CHO-K1 cells containing GRP10 was performed using the calcium-sensitive dye Fura-2 according to the manufacturer’s protocol (Molecular Devices, Sunnyvale, CA, USA). The day before the experiment, cells were seeded at 40,000 cells/well in 96-well plates in growth media and kept at 37 °C in an incubator with 5% CO_2_ overnight. Peptides were tested at concentrations from 10^−12^ to 10^−5^ M. Fura-2 fluorescent dye was detected using a FlexStation 3 fluorometric plate reader (Molecular Devices), and excitation was measured at 340 nm and 380 nm and emission at 510 nm.

The intracellular Ca^2+^ level was measured using the AequoScreen stable CHO-K1 cell line containing NPFF-R2 purchased from Perkin Elmer according to the manufacturer’s protocol. Cells at 80–90% confluence cultured in media without selective antibiotics were detached (PBS pH 7.4 + 0.5 mM EDTA) and centrifuged. Cells resuspended in phenol red-free DMEM with 0.1% protease-free BSA and 5 µM coelenterazine h (Thermo Fisher Scientific Inc. Brand) were seeded at 50,000 cells/well in 96-well plates and incubated in the dark at RT with gentle agitation for 4 h. Peptides were tested at concentrations from 10^−12^ to 10^−5^ M. Luminescent light emission was recorded using a FlexStation 3 plate reader.

### 4.8. Cell Signaling Determined Using Beta-Lactamase Reporter System

Cell lines containing beta-lactamase reporter genes with different receptors, GPR10, Y_5_, GHSR and opioid receptors, were used to study the agonist/antagonist properties of PrRP31 and lipidized PrRP31 analogs. Cells were seeded at 10,000 cells/well in a 384-well plate in assay medium, and the assay was performed according to Thermo Fisher’s protocol and according to our previous study [10]. Receptor agonists were tested at concentrations from 10^−12^ to 10^−5^ M. The concentration of the agonist in antagonist assay mode ranged from 10^−12^ to 10^−5^ M, and the potential antagonists PrRP31 and palm^11^-PrRP31 were tested at concentrations from 10^−7^ or 10^−6^ to 10^−5^ M. Fluorescence was detected at 409 nm excitation and 460 and 530 nm emissions using the FlexStation 3 fluorometric plate reader.

### 4.9. Statistical Analysis

Data were analyzed by GraphPad Software (San Diego, CA, USA) and are presented as the means ± SEM. The saturation and competitive binding experiments were analyzed according to [46] using the Cheng-Prusoff equation [33]. The competitive binding curves were plotted compared to the best fit for single-binding site models, and half maximal inhibitory concentration (IC_50_) values were obtained from nonlinear regression analysis. From saturation binding experiments, the dissociation constant (K_d_) and number of binding sites/cell (Bmax) were calculated. Inhibition constants (K_i_) were calculated from IC_50_ values, K_d_ and the concentration of radioligands.

Experiments using immunoblotting were analyzed using one-way ANOVA followed by Dunnett’s post hoc test; *p* < 0.05 was considered statistically significant. Dose-response curves were obtained from nonlinear regression.

The beta-lactamase assay results were analyzed by nonlinear regression as log agonist versus response, and EC_50_ values were determined in agonist mode using GraphPad software. Data are representative of at least two experiments, each performed in duplicate.

Ca^2+^ release assay data are shown as the percentage of maximal response, and the results were analyzed by nonlinear regression as log agonist versus response using GraphPad software. Data are representative of at least three experiments, each performed in duplicate.

## 5. Conclusions

Lipidized PrRP31 analogs have great potential for the treatment of obesity and neurodegenerative diseases. The in vitro properties of the two most potent palmitoylated analogs, palm-PrRP31 and palm^11^-PrRP31, were tested and compared. Palmitoylation of PrRP31 increased not only the activity and binding affinity to GPR10 and NPFF-R2, which are both connected with food intake regulation, but also the binding properties and activity to NPFF-R1. Therefore, NPFF-R1 is a new target of lipidized PrRP31 analogs. Both analogs activated the cellular signaling of the PKB/Akt and MAPK pathways and activated the transcription factors c-Fos, c-Jun and CREB in cells expressing GPR10 and NPFFR-2. Activation of all previously mentioned cellular pathways in cells expressing NPFF-R1 was observed only after incubation with palm-PrRP31. Palm-PrRP31 also showed higher off-target activity on GHSR receptors and Y receptors than palm^11^-PrRP31; therefore, the more selective palm^11^-PrRP31 has a better potential for obesity treatment. Our future studies will focus on further development of palmitoylated PrRP analogs with minimized off-target activity.

## Figures and Tables

**Table 1:** Structures of human prolactin-releasing peptide 31 (PrRP31), neuropeptide FF (NPFF) and its analogs.

Analog	Sequence
PrRP31	SRAHQHSMETRTPDINPAWYTGRGIRPVGRF-NH_2_
Palm^11^-PrRP31	SRTHRHSMEIK(γ-E (N-palm))TPDINPAWYASRGIRPVGRF-NH_2_
Palm-PrRP31	(N-palm)SRTHRHSMEIRTPDINPAWYASRRGIRPVGRF-NH_2_
NPFF	FLFQPQRF-NH_2_
1DMe	yL(N-Me)FQPQRF-NH_2_

**Table 2:** Binding affinities of natural PrRP31, its analogs and other peptides to tested receptors.

Receptor	GPR10	NPFF-R2	NPFF-R1	KOR
	[^125^I]-PrRP31	[^125^I]-1DMe	[^125^I]-1DMe	[^125^I]-Dynorphin
	K_i_ [nM]			
PrRP31	4.58 ± 0.66	26.73 ± 9.01	40.39 ± 4.20	>10,000
Palm^11^-PrRP31	3.44 ± 0.36	7.66 ± 1.33	13.52 ± 1.57	4278 ± 866
Palm-PrRP31	4.04 ± 0.01	0.77 ± 0.19	0.78 ± 0.11	106 ± 15
NPFF	>10,000	0.28 ± 0.06	1.08 ± 0.09	-
1DMe	>10,000	1.03 ± 0.23	0.79 ± 0.06	-
Dynorphin	-	-	-	0.36 ± 0.03
**Receptor**	**Y_1_**	**Y_2_**	**Y_5_**	**GHSR**
	**[^125^I]-PYY**	**[^125^I]-PYY**	**[^125^I]-PYY**	**[^125^I]-Ghrelin**
	**K_i_ [nM]**			
PYY	2.92 ± 0.28	6.51 ± 0.71	3.06 ± 0.49	-
PrRP31	>10,000	>10,000	2863 ± 43	>10,000
Palm^11^-PrRP31	>10,000	>10,000	362 ± 96	2800 ± 466
Palm-PrRP31	3147 ± 31	>10,000	32.62 ± 6.16	160 ± 16
Ghrelin	-	-	-	4.59 ± 0.41

- not determined; data presented as the means K_i_ values ± SEM and analyzed in Graph-Pad Software were performed in 2–5 independent experiments in duplicates. K_i_ was calculated using the Cheng-Prusoff equation [33].

**Table 3:** Summary table of signaling pathways tested using immunoblot in cells expressing GPR10, NPFF-R2 and NPFF-R1.

	PrRP31			Palm^11^-PrRP31			Palm-PrRP31			NPFF			1DMe		
Receptor	GPR10	NPFF-R2	NPFF-R1	GPR10	NPFF-R2	NPFF-R1	GPR10	NPFF-R2	NPFF-R1	GPR10	NPFF-R2	NPFF-R1	GPR10	NPFF-R2	NPFF-R1
p-ERK	**↑** **	**↑** **	-	**↑** ***	**↑** ***	**↑** ***	**↑** ***	**↑** ***	**↑** ***	-	**↑** ***	**↑** ***	**↑** *	**↑** ***	**↑** ***
p-JNK	**↑** *	**↑** **	-	**↑** ***	**↑** ***	-	**↑** ***	**↑** ***	**↑** **	-	**↑** **	**↑** ***	**↑** *	**↑** **	**↑** ***
p-p38	**↑** **	-	-	**↑** ***	**↑** *	-	**↑** ***	**↑** **	**↑** ***	-	-	**↑** *	-	**↑** **	**↑** ***
p-Akt S473	**↑** *	**↑** ***	-	**↑** ***	**↑** ***	**↑** *	**↑** ***	**↑** **	**↑** **	-	**↑** ***	**↑** ***	**↑** *	**↑** ***	**↑** ***
p-Akt T308	-	**↑** ***	-	**↑** ***	**↑** ***	**↑** ***	**↑** *	**↑** ***	**↑** ***	-	**↑** ***	**↑** ***	-	**↑** ***	**↑** ***
p-PKA	-	-	-	-	-	-	-	-	-	-	-	-	-	-	-
c-Jun	**↑** **	**↑** **	-	**↑** ***	**↑** ***	-	**↑** **	**↑** ***	**↑** ***	-	**↑** ***	**↑** **	-	**↑** ***	**↑** ***
c-Fos	**↑** **	**↑** *	-	**↑** **	**↑** ***	-	**↑** ***	**↑** ***	**↑** ***	-	**↑** **	**↑** *	-	**↑** ***	**↑** ***
p-CREB	**↑** **	**↑** *	-	**↑** ***	**↑** *	-	**↑** ***	**↑** *	**↑** **	-	**↑** **	-	-	**↑** *	-

**↑** significant activation (* *p* < 0.05, ** *p* < 0.01, *** *p* < 0.001), - no significant changes.

**Table 4:** Agonist properties on GPR10 and other potential off-target receptors determined using β-lactamase assay.

Receptor	GPR10	Y_5_	GHSR	KOR	DOR	MOR	ORL-1
	EC_50_ [pM]	EC_50_ [nM]					
PrRP31	530.3 ± 70.5	N	N	N	N	N	N
PYY		19.4 ± 2.5					
Ghrelin			2.8 ± 2.5				
U-50488				1.4 ± 1.0			
Deltorphin II					5.6 ± 9.9		
DAMGO						14.7 ± 1.9	
Nociceptin							3.8 ± 0.6
Palm^11^-PrRP31	39.1 ± 5.1	583.3 ± 121.1	1068.1 ± 272.2	>10,000	N	N	N
Palm-PrRP31	71.8 ± 6.4	56.5 ± 18.4	1273.5 ± 167.9	>10,000	N	N	N

Data presented as the means EC_50_ values ± SEM and analyzed in Graph-Pad Software and performed in 2–3 independent experiments in duplicates; N-no agonist properties.

**Table 5:** Primary antibodies used for immunoblotting and their dilutions.

Antibody Against	Source	Dilution
Phospho-Akt (Thr308) (#2965)	Rabbit	1:1000, 5% BSA, TBS/T-20
Phospho-Akt (Ser473) (#4060) Akt (#4691S)	Rabbit Rabbit	1:1000, 5% BSA, TBS/T-20 1:1000, 5% BSA, TBS/T-20
Phospho-CREB (Ser133) (#9196) CREB (#9104S)	Mouse Mouse	1:1000, 5% milk, TBS/T-20 1:1000, 5% milk, TBS/T-20
Phospho-p44/42 MAPK (Erk1/2) (Thr202/Tyr204) (#4370S) p44/42 MAPK (Erk1/2) (#9107S)	Rabbit Mouse	1:2000, 5% BSA, TBS/T-20 1:2000, 5% milk, TBS/T-20
Phospho-SAPK/JNK (Thr183/Tyr185) (#4668) SAPK/JNK (#9252)	Rabbit Rabbit	1:1000, 5% BSA, TBS/T-20 1:1000, 5% BSA, TBS/T-20
Phospho-p38 MAPK (Thr180/Tyr182) (#4511) p38 MAPK (#9212)	Rabbit Rabbit	1:1000, 5% BSA, TBS/T-20 1:1000, 5% BSA, TBS/T-20
Phospho-PKA C (Thr197) (#5661)	Rabbit	1:1000, 5% BSA, TBS/T-20
c-Fos (#2250)	Rabbit	1:1000, 5% BSA, TBS/T-20
c-Jun (#9165)	Rabbit	1:1000, 5% BSA, TBS/T-20
GAPDH (#97166)	Mouse	1:1000, 5% milk, TBS/T-20

## Data Availability

Not applicable.

## Supplementary Materials

The following are available online at https://www.mdpi.com/article/10.3390/ijms22168904/s1, Supplementary Figure S1: Induction of (A) PKA phosphorylation after 5 min incubation at 37 °C with peptides in final concen-18 trations 10-6 M in CHO-K1 cells expressing receptors GPR10, NPFF-R2 and NPFF-R1, Supplementary Figure S2: Antagonist mode of FRET assay showing effect of PrRP31 and palm11-PrRP31 at (A) opioid receptors and 23 (B) GHSR.
