# Supplementary material for: Palmitoylation of Prolactin-Releasing Peptide Increased Affinity for and Activation of the GPR10, NPFF-R2 and NPFF-R1 Receptors: In Vitro Study
Source: Int J Mol Sci. 2021 Aug 18;22(16):8904. doi: 10.3390/ijms22168904 (PMC8396344; doi:10.3390/ijms22168904)
Supplement: Supplementary file 1 [file ijms-22-08904-s001.zip › ijms-1303643-supplementary.pdf]

International Journal of
Molecular Sciences

Supllementary data

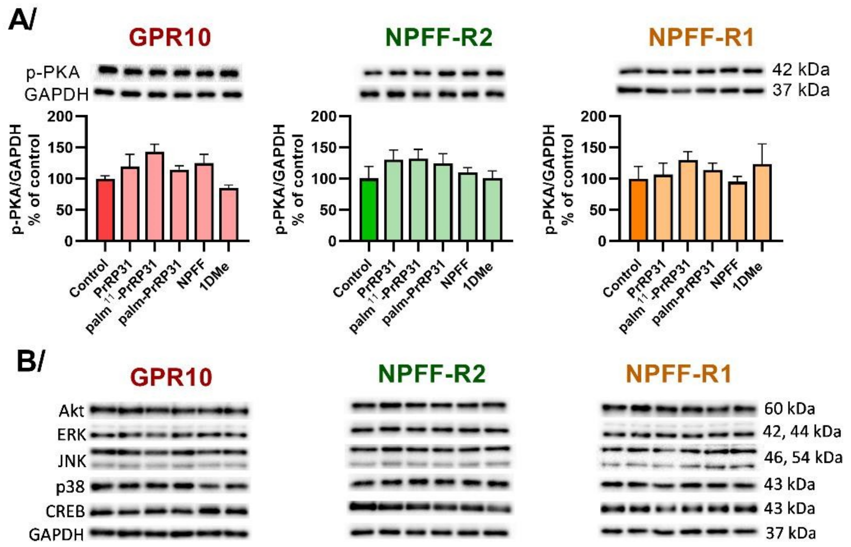

Supplementary Figure S1. Induction of **(A)** PKA phosphorylation after 5 minutes incubation at 37°C with peptides in final concentrations 10-6 M in CHO-K1 cells expressing receptors GPR10, NPFF-R2 and NPFF-R1. Densitometric quantification was normalized to GAPDH and the phosphorylation level in the untreated control was standardized as 100% **(B)** total protein levels of tested signaling pathways.

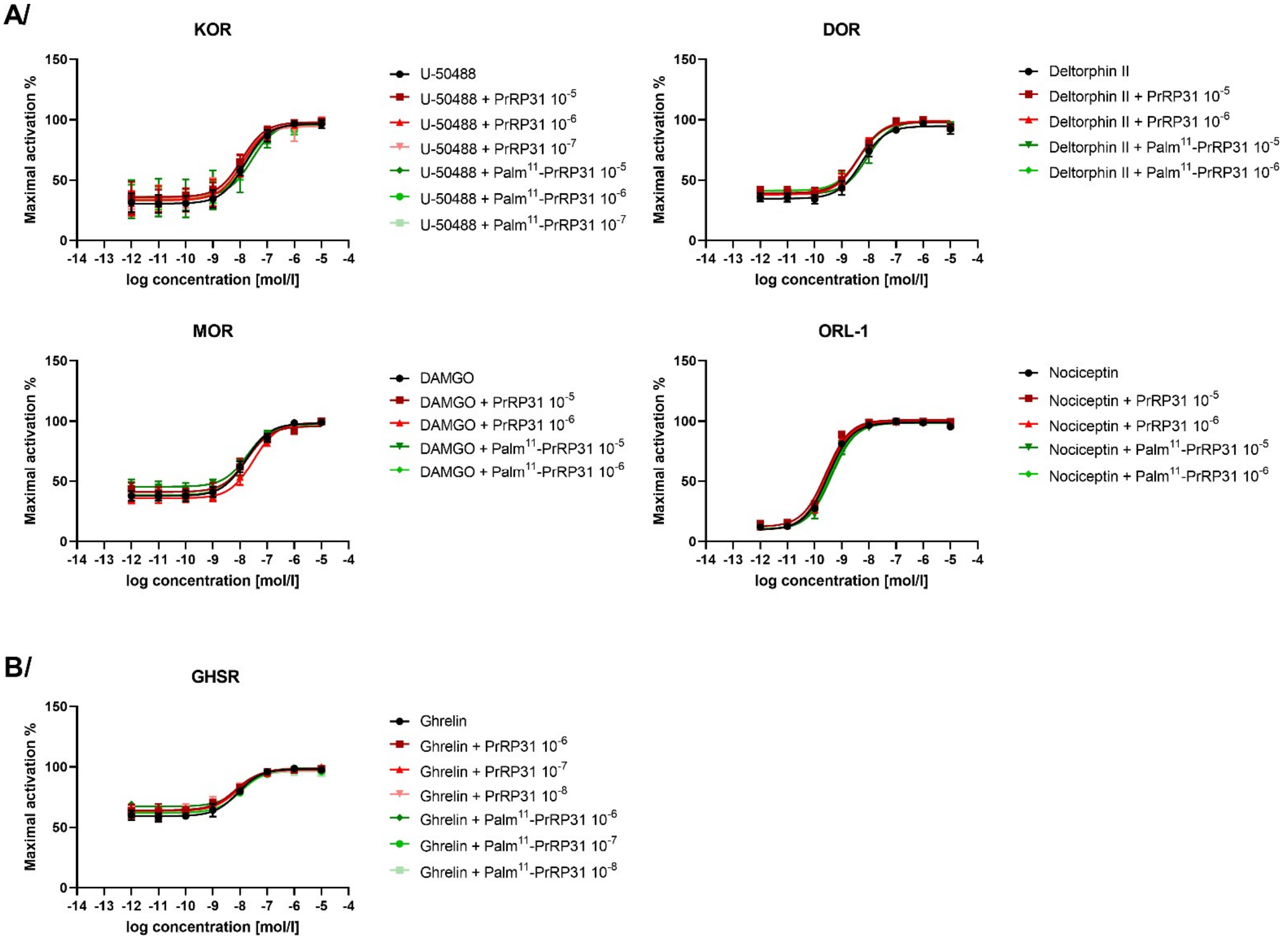

Supplementary Figure S2. Antagonist mode of FRET assay showing effect of PrRP31 and palm^{11}-PrRP31 at **(A)** opioid receptors and **(B)** GHSR. Data are presented as mean ± SEM and the experiments were performed in duplicates and repeated at least two times and analyzed using nonlinear regression
